# The complete chloroplast genome of *Eria corneri* (Orchidaceae)

**DOI:** 10.1080/23802359.2019.1692734

**Published:** 2019-11-21

**Authors:** Shan-Hu Ma, Juan Chen, Jie Zhou, Qing-Dong Zheng, Ming-Kun Chen, Tai-Xiang Xie, Ye Ai

**Affiliations:** Key Laboratory of National Forestry and Grassland Administration for Orchid Conservation and Utilization, College of Landscape Architecture, Fujian Agriculture and Forestry University, Fuzhou, China

**Keywords:** Chloroplast genome, phylogenetic analysis, Orchidaceae, *Eria corneri*

## Abstract

*Eria corneri* is a perennial epiphytic orchid distributed in southeastern China with high value of ornamental and medicinal. In this study, the complete chloroplast genome sequence of *E. corneri* was determined from Illumina pair-end sequencing data. The complete chloroplast genome sequence of *E. corneri* is 150,538 base pairs (bp) in length, including one large single-copy region (LSC, 85,941 bp), one small single-copy region (SSC, 13,099 bp), and a pair of inverted repeat regions (IRs) of 25,749 bp. Besides, the complete chloroplast genome contains 132 genes, including 77 protein-coding genes, 38 tRNA genes, and 8 rRNA genes. Phylogenetic analysis showed that *E. corneri* was most closely related to *Calanthe triplicata* and *Calanthe davidii*. Our study provides a foundation for the identification and genotyping of *Eria* species.

The genus *Eria* is regarded as one of the largest genera in Orchidaceae family, with approximately 370 species worldwide, distributed in tropical Asia to Oceania, and there are 43 species in China, produced in southeastern provinces (Chen et al. 1999; Pridgeon et al. 2009). *E. corneri* is a perennial epiphytic orchid that grows on trees or rocks at an altitude of 500 to 1500 meters (Chen et al. 1999). Pharmacological researches showed that some chemical constituents of *E. corneri*, such as erianin and confusarin, have good antitumor and anti-oxidation activities (Bhuiya et al. 2017). In this study, we report the complete chloroplast genome of *E. corneri*. On the one hand, it will contribute to the species identification, germplasm diversity, and genetic engineering of the *Eria* genus (Lin et al. 2015). On the other hand, this is of great significance to the research and development of *E. corneri* at the genetic level.

The samples were collected from Qinglong Waterfall Scenic Area, Yongtai County, Fuzhou City, Fujian province, China (25°42′46.14′´N, 118°50′12.68″E), and deposited at Herbarium of Fujian Agriculture and Forestry University (specimen code: YT-BZML). The genomic DNA was extracted from fresh leaves using a modified CTAB method (Doyle and Doyle 1987) and sequenced by the BGISEQ-500 platform. The clean reads were used to assemble the complete chloroplast genome by the GetOrganelle pipe-line (Jin et al. 2018), with the chloroplast genome of *Liparis nervosa* as the reference sequences. The assembled chloroplast genome was annotated using the Geneious R11.15 (Kearse et al. 2012). Finally, we obtained a complete chloroplast genome of *E. corneri* and submitted to GenBank with accession number MN477202.

The complete chloroplast genome of *E. corneri* is 150,538 bp in length, containing a large single-copy (LSC) region of 85,941 bp, a small single-copy (SSC) region of 13,099 bp, and two inverted repeat (IR) regions of 25,749 bp. The new sequence has a total of 132 genes, including 77 protein-coding genes, 38 tRNA genes, and 8 rRNA genes. The overall GC-content of the whole plastome is 43.3%, whereas the corresponding values of the LSC, SSC, and IR regions are 57.09%, 8.70%, and 17.1%, respectively.

To confirm the phylogenetic position of *E. corneri*, a molecular phylogenetic tree was constructed based on 13 complete chloroplast genome sequences of Orchidaceae (*Calanthe triplicata*, *Calanthe davidii*, *E. corneri, Cattleya crispata*, *Masdevallia coccinea*, *Corallorhiza macrantha*, *Pelatantheria scolopendrifolia*, *Gastrochilus fuscopunctatus*, *Neofinetia falcata*, *Thrixspermum japonicum*, *Phalaenopsis equestris*, *Erycina pusilla*, *Cymbidium faberi*). All sequences were aligned with the HomBlock pipeline (Bi et al. 2018) and subsequently checked manually in Bioedit v5.0.9 (Hall 1999). Then, the phylogenetic tree constructed by RAxML (Stamatakis 2014) with 1000 ultrafast bootstrap (UFBoot) replicates (Minh et al. 2013; Chernomor et al. 2016). The results showed that *E. corneri* was mostly related to *Calanthe triplicata* and *Calanthe davidii* (Figure 1).

**Figure 1.**
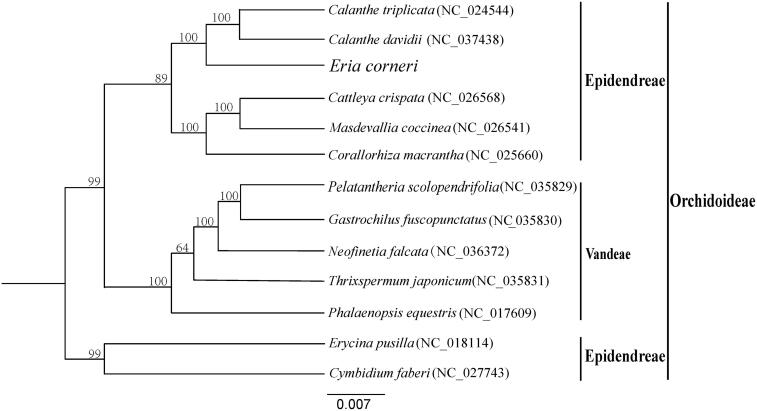
A phylogenetic tree was constructed based on 13 complete chloroplast genome sequences of Orchidaceae. All the sequences were downloaded from NCBI GenBank.
